# Comparison of ischemic cardiovascular events between dapagliflozin and empagliflozin in combination with metformin: A nationwide population-based cohort study

**DOI:** 10.1371/journal.pone.0333604

**Published:** 2025-10-16

**Authors:** Hayeon Kim, Seung Won Lee, Yejee Lim, Nayoung Han, Suin Kang, Youngjoo Byun, Kyungim Kim

**Affiliations:** 1 College of Pharmacy, Korea University, Sejong, Republic of Korea; 2 Institute of Pharmaceutical Science, Korea University, Sejong, Republic of Korea; 3 Department of Internal Medicine, Seoul National University Bundang Hospital, Seoul National University College of Medicine, Seongnam, Republic of Korea; 4 College of Pharmacy and Research Institute of Pharmaceutical Sciences, Jeju National University, Jeju, Republic of Korea; 5 Education and Research Group for the Convergence of New Approach Methodologies and Innovative Drug Development, Korea University, Sejong, Republic of Korea; University of Diyala College of Medicine, IRAQ

## Abstract

The comparative effectiveness of individual sodium–glucose cotransporter-2 inhibitors (SGLT-2is) in preventing ischemic cardiovascular disease (CVD) remains uncertain. Thus, this study compared the incidence of ischemic CVD events in patients with type 2 diabetes mellitus (T2DM) treated with dapagliflozin or empagliflozin in combination with metformin. This retrospective cohort study analyzed national claims data from the Korean National Health Insurance Service. Patients with T2DM who received dapagliflozin or empagliflozin, combined with metformin, between 2014 and 2019 were included. The primary outcome was composite ischemic CVD events, defined as myocardial infarction, ischemic stroke, or coronary revascularization. Secondary outcomes included each component of composite ischemic CVD events, unstable angina, and all-cause mortality. Hazard ratios (HRs) and confidence intervals (CIs) were estimated using Cox proportional hazards models, adjusting for covariates in three stepwise models: Model 1 (age and sex), Model 2 (Model 1 variables plus patient characteristics), and Model 3 (Model 2 variables plus clinical parameters). In Model 3, after full adjustment for systolic blood pressure, low-density lipoprotein cholesterol, fasting blood glucose, and serum creatinine, no significant difference was observed in the incidence of composite ischemic CVD events between dapagliflozin and empagliflozin when each was used in combination with metformin (adjusted HR 0.50, 95% CI: 0.24–1.03). Additionally, no significant differences were observed in individual components of composite ischemic CVD events, unstable angina, and all-cause mortality. These real-world findings may help in selecting an SGLT-2is subtype for CVD prevention in Asian patients with T2DM.

## Introduction

Type 2 diabetes mellitus (T2DM) is a representative chronic disease and a significant public health concern, placing a substantial burden not only on individuals but also on society. Globally, the age-standardized incidence rate of T2DM was 184.6 per 100,000 people in 2017 and is projected to rise to 284.4 per 100,000 people in the 2030s [1]. Diabetes is also associated with a reduction in life expectancy by approximately 10 years [2], and two-thirds of deaths among patients with T2DM are attributed to cardiovascular disease (CVD) [3]. Macrovascular disease, particularly of cardiac origin, is a common complication of T2DM. Studies have shown that patients with T2DM have a 1.5 to 2.3 times higher risk of cardiovascular death than the nondiabetic population [4–6]. Therefore, current T2DM management guidelines emphasize not only blood glucose control but also strategies for CVD prevention [7].

Sodium-glucose cotransporter-2 inhibitors (SGLT-2is) have shown significant clinical benefits for CVD in patients with T2DM. Large-scale randomized controlled trials (RCTs) and real-world studies have confirmed the positive effects of SGLT-2is on key cardiovascular outcomes, including major adverse cardiovascular events, cardiovascular death, and hospitalization for heart failure [8–10]. Consequently, there is a growing interest in investigating whether different SGLT-2is subtypes have varying effects on reducing the risk of CVD. In previous RCTs, varying degrees of effects in reducing CVD risk have been reported for two widely prescribed SGLT-2is, dapagliflozin and empagliflozin [8,11,12]. However, no RCTs have directly compared the two SGLT-2is. Some network meta-analyses have evaluated their differences in reducing CVD risk, but the findings remain inconclusive [13–16]. Furthermore, these studies have limitations as they combined data from both RCTs and observational studies or relied on indirect analyses mediated using a placebo. Several retrospective cohort studies have compared the risk of CVD between dapagliflozin and empagliflozin [17–21]. However, these cohort studies did not restrict the background antidiabetic agent class, and the study population received various antidiabetic combinations, making it difficult to isolate SGLT-2is-specific effects. Because the extent of antidiabetic agent use often reflects diabetes severity, a key confounder for CVD, the homogeneity of the underlying antidiabetic regimens is essential [22,23]. Moreover, certain antidiabetic agents may directly affect CVD risk [24–26], further complicating the interpretation of study results.

While dapagliflozin and empagliflozin show pharmacokinetic (PK) and pharmacodynamic (PD) differences, there is no evidence for a clinically meaningful difference in glycemic control; thus, agent selection can be individualized by comorbidities [27–30]. In this context, understanding differences in CVD risk reduction across SGLT-2is subtypes may help tailor their use in clinical practice. Therefore, this study aimed to compare the risk of subsequent ischemic CVD events in adults with T2DM who initiated dapagliflozin or empagliflozin, both combined with metformin, using nationwide claims data from South Korea. Previous studies have shown that metformin, the first-line antidiabetic agent commonly used in combination with SGLT-2is in clinical practice, has no effect on CVD incidence [31,32]. Moreover, it has been reported that combination of metformin with either empagliflozin or dapagliflozin exhibits no clinically meaningful differences in glucose-lowering effects or serious adverse effects, regardless of which SGLT-2i is used [29,30]. Therefore, the present study restricted baseline antidiabetic agents to metformin to better reflect real-world practice, ensure homogeneity of the study group, and enable a clearer comparison of the effects of SGLT-2is.

## Materials and methods

### Data source

This retrospective, nationwide, population-based cohort study was conducted using customized data from the National Health Insurance Service (NHIS) claims data of South Korea. The NHIS is a national healthcare insurance system covering 97% of the Korean population. Its claims database provides anonymized, longitudinal health data, including sociodemographic information, medical diagnoses (coded using the International Classification of Diseases, Tenth Revision [ICD-10]), therapeutic procedures, drug prescriptions (prescription date, supply duration, dosage, and administration route), and healthcare use type (outpatient, inpatient, or emergency department). The structure of the NHIS data has been detailed in a previous publication [33]. The NHIS also provides health checkup data, including physical examination results (height, weight, and blood pressure); selected laboratory test findings (low-density lipoprotein cholesterol [LDL-C], fasting blood glucose [FBG], serum creatinine [SCr], total cholesterol, triglycerides, high-density lipoprotein cholesterol, hemoglobin, liver function tests, and urinalysis); and information on personal and family medical history, and health-related behaviors, such as smoking status and alcohol consumption. These data are collected through annual or biennial general health checkups for NHIS-insured individuals and their dependents. For this study, both the claims and the health checkup data from the NHIS were used.

The study was approved by the Institutional Review Board of Korea University (KUIRB-2021-0036-01) and the Korea NHIS National Health Information Data Request Review Committee (NHIS 2021-1-410). The requirement for informed consent was waived because NHIS provided anonymized data. All data used in this study were fully anonymized before being accessed by the researchers. Therefore, the authors had no access to any information that could identify individual patients during and after data collection. Data used for this study were accessed for research purposes from March 24, 2021, to March 23, 2023. This study followed the Strengthening the Reporting of Observational Studies in Epidemiology (STROBE) reporting guideline [34].

### Study population

**Fig 1:**
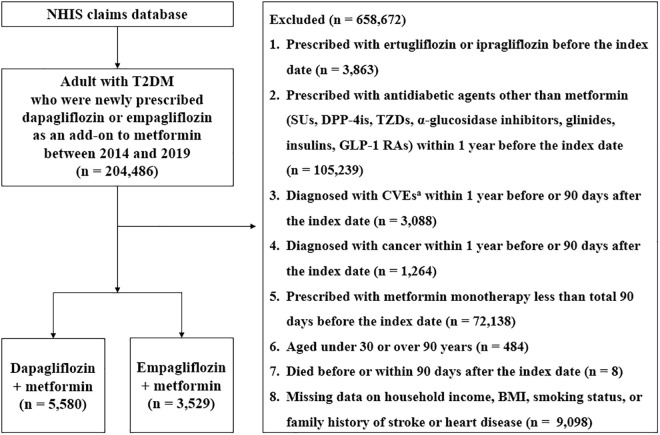
Flow diagram of the study population. This cohort study used customized Korean claims data from the NHIS database. Patients with T2DM who newly initiated dapagliflozin or empagliflozin as an add-on to metformin between 2014 and 2019 were identified. After applying predefined exclusion criteria, 9,109 eligible patients remained, including 5,580 treated with dapagliflozin and 3,529 with empagliflozin. ^a^CVEs include ischemic heart diseases (I20–I25), coronary revascularization (procedure codes: M6551, M6552, M6561, M6563, M6564, M6571, M6572, O1641, O1642, O1647, OA641, OA642, and OA647), heart failure (I50), cerebrovascular disease (I60–I69), or transient cerebral ischemic attack (G45). Abbreviations: BMI, body mass index; CVEs, cardiovascular events; DPP-4is, dipeptidyl peptidase-4 inhibitors; GLP-1RAs, glucagon-like peptide-1 receptor agonists; SUs, sulfonylureas; T2DM, type 2 diabetes mellitus; TZDs, thiazolidinediones.

From the NHIS database, which includes approximately 100 million individuals, this study identified adults aged 30–90 years with T2DM who were newly prescribed dapagliflozin or empagliflozin as an add-on to metformin between 2014 and 2019. Patients with T2DM were defined as individuals who met the following two criteria: a primary or subdiagnosis of T2DM (ICD-10 code E11) and at least one prescription history for an antidiabetic agent. This operational definition was validated in a Korean study using NHIS claims data with high specificity and accuracy and is consistent with Canadian validation findings showing that diagnosis-plus-prescription algorithms achieve very high specificity [35,36]. Patients were included if they had received metformin monotherapy for at least 90 days before adding SGLT-2is to minimize the possibility of metformin-induced glycemic fluctuations. This 90-day duration was selected based on previous studies indicating that metformin provides no statistically significant additional blood glucose–lowering effects after approximately 3 months (90 days) [37,38]. The initiation date of dapagliflozin or empagliflozin was designated as the index date. To ensure comparability between the treatment groups and assess the actual effects of each SGLT-2i, this study excluded patients treated with antidiabetic agents other than metformin within 1 year before the index date. Additionally, individuals who had previously used other subtypes of SGLT-2is (such as ertugliflozin or ipragliflozin) before the index date were also excluded. To minimize the impact of pre-existing CVD events or cancer on the study outcomes, individuals with a history of ischemic heart disease (I20–I25), heart failure (I50), cerebrovascular disease (I60–I69), coronary revascularization (percutaneous coronary intervention or coronary artery bypass graft), or transient cerebral ischemic attack (G45) were excluded. Patients diagnosed with any cancer type within 1 year before or 90 days after the index date were also excluded. The exclusion criterion for pre-existing CVD events was based on evidence suggesting that most recurrent CVD events tend to occur within the first year following the initial episode [39,40]. The study selection process is presented in Fig 1.

### Study outcomes

The primary outcome was composite ischemic CVD events, defined as a patient hospitalized or visited the emergency department for myocardial infarction (MI) (I21–I23), coronary revascularization procedures (M6551, M6552, M6561, M6563, M6564, M6571, M6572, O1641, O1642, O1647, OA641, OA642, OA647), or ischemic stroke (I63). The secondary outcomes included individual components of the composite ischemic CVD events, unstable angina (I20.0), and all-cause mortality. Study outcomes were determined based on at least one record of hospitalization or emergency department visit with the corresponding diagnostic or procedure code as the primary or first subdiagnosis. Patients were followed from 90 days after the index date until the first occurrence of the study outcome, death from any cause, or the end of the study (December 31, 2019), whichever occurred first. The 90-day lag period was prespecified to account for the induction/latency period required for SGLT-2is to affect ischemic CVD risk and to reduce reverse causation. This design is supported by large-scale RCTs on dapagliflozin and empagliflozin, which showed that cardiovascular benefits typically emerged approximately 3 months after initiation [11,12,41]. The same lag was applied to both study groups to minimize differential immortal time [42].

### Covariates

The study covariates included age, sex, metformin monotherapy duration, index year, household income, region of residence, Charlson comorbidity index (CCI), comorbidities, comedications, and health checkup data. The duration of metformin monotherapy was defined as the period during which metformin was used alone before adding dapagliflozin or empagliflozin, starting in 2013. CCI scores were calculated based on patients’ disease records using previously validated algorithms [43]. Comorbidities included hypertension, dyslipidemia, atrial fibrillation, chronic kidney disease, microvascular complications of diabetes, and rheumatoid arthritis, all diagnosed within 1 year before the index date. Comedications were defined as antihypertensive, antihyperlipidemic, antiplatelet, and anticoagulant agents prescribed for more than total 90 days within 1 year before the index date. The relevant ICD-10 codes and generic names for comorbidities and comedications are provided in S1 and S2 Tables. Health checkup data recorded within 2 years before the index date were included as covariates. These variables comprised body mass index (BMI), smoking status, family history of stroke or heart disease (MI or angina pectoris), systolic blood pressure (SBP), LDL-C, FBG, and SCr.

### Statistical analyses

Baseline characteristics of the dapagliflozin and empagliflozin groups were presented as frequencies and percentages and compared using the χ^2^ test. Cox proportional hazards regression models were used to estimate the hazard ratios (HRs) and 95% confidence intervals (CIs) for the risk of composite ischemic CVD events in the empagliflozin group relative to the dapagliflozin group. Crude and adjusted HR (aHR) were estimated for individual components of composite ischemic CVD events, unstable angina, and all-cause mortality. The covariates were adjusted in three sequential models to show the incremental impact of broader baseline information on the effect estimates: Model 1 adjusted for age and sex; Model 2 adjusted for the variables in Model 1 as well as metformin monotherapy duration, index year, comorbidities, CCI score, comedications, BMI, smoking status, and family history of stroke or heart disease; and Model 3 adjusted for the variables in Model 2 along with specific clinical parameters from health checkup data (SBP, LDL-C, FBG, and SCr) measured within 2 years before the index date. Because Model 3 represented the fully adjusted and most conservative specification, it served as the primary basis for inference.

Subgroup analyses were conducted to assess crude HR and aHR in Model 2 for composite ischemic CVD events, stratified by age group, sex, BMI, FBG, estimated glomerular filtration rate (eGFR), and LDL-C. However, due to the small sample size in each subgroup in Model 3, further subgroup analyses for Model 3 were not conducted. All analyses followed an intention-to-treat approach and were conducted using SAS software, Version 9.4 (SAS Institute Inc., Cary, NC, USA). A two-sided *p*-value of <0.05 was considered statistically significant.

## Results

### Study population and baseline characteristics

This study included 9,109 patients who were newly prescribed SGLT-2is as an add-on to metformin, with 5,580 receiving dapagliflozin and 3,529 receiving empagliflozin (Fig 1). Detailed baseline characteristics are presented in Table 1. The mean follow-up duration was 747.6 ± 495.9 days for the dapagliflozin group and 529.5 ± 341.9 days for the empagliflozin group. The mean age was 52.82 ± 9.78 for dapagliflozin group and 53.86 ± 9.83 for empagliflozin group.

**Table 1:** Baseline characteristics of dapagliflozin and empagliflozin groups.

Variables	Models 1 and 2			Model 3		
	DAPA + MET (n = 5,580)	EMPA + MET (n = 3,529)	*p*	DAPA + MET (n = 4,203)	EMPA + MET (n = 2,391)	*p*
Sex			0.33			0.15
Men	3,226 (57.81)	2,077 (58.86)		2,406 (57.24)	1,412 (59.05)	
Women	2,354 (42.19)	1,452 (41.14)		1,797 (42.76)	979 (40.95)	
Age (years)			<0.001			0.04
30–39	462 (8.28)	270 (7.65)		341 (8.11)	184 (7.70)	
40–49	1,654 (29.64)	920 (26.07)		1,267 (30.15)	648 (27.10)	
50–59	2,097 (37.58)	1,322 (37.46)		1,602 (38.12)	924 (38.64)	
60–69	1,102 (19.75)	822 (23.29)		800 (19.03)	518 (21.66)	
70–79	239 (4.28)	180 (5.10)		172 (4.09)	108 (4.52)	
80–89	26 (0.47)	15 (0.43)		21 (0.50)	9 (0.38)	
Metformin monotherapy duration (days)	572.17 ± 437.33	640.66 ± 464.99	<0.001	533.45 ± 400.20	608.44 ± 431.74	<0.001
Index year			<0.001			<0.001
2014	103 (1.85)	0 (0.00)		100 (2.38)	0 (0.00)	
2015	535 (9.59)	0 (0.00)		511 (12.16)	0 (0.00)	
2016	843 (15.11)	251 (7.11)		797 (18.96)	238 (9.95)	
2017	1,131 (20.27)	805 (22.81)		1,052 (25.03)	734 (30.70)	
2018	1,423 (25.50)	1,111 (31.48)		1,098 (26.12)	872 (36.47)	
2019	1,545 (27.69)	1,362 (38.59)		645 (15.35)	547 (22.88)	
Household income^a^			0.28			0.62
Low (0–5)	1,048 (18.78)	717 (20.32)		780 (18.56)	472 (19.74)	
Medium–low (6–10)	975 (17.47)	626 (17.74)		730 (17.37)	419 (17.52)	
Medium–high (11–15)	1,396 (25.02)	859 (24.34)		1,042 (24.79)	591 (24.72)	
High (16–20)	2,161 (38.73)	1,327 (37.60)		1,651 (39.28)	909 (38.02)	
Region of residence^b^			0.80			0.84
Urban	2,473 (44.32)	1,554 (44.04)		1,865 (44.37)	1,054 (44.08)	
Rural	3,107 (55.68)	1,975 (55.96)		2,338 (55.63)	1,337 (55.92)	
Comorbidity						
Hypertension	2,546 (45.63)	1,700 (48.17)	0.02	1,942 (46.21)	1,128 (47.18)	0.46
Dyslipidemia	3,041 (54.50)	1,949 (55.23)	0.50	2,322 (55.25)	1,329 (55.58)	0.80
Atrial fibrillation	59 (1.06)	29 (0.82)	0.27	36 (0.86)	19 (0.79)	0.89
Chronic kidney disease	16 (0.29)	13 (0.37)	0.57	10 (0.24)	9 (0.38)	0.34
Microvascular complications^c^	737 (13.21)	495 (14.03)	0.27	569 (13.54)	350 (14.64)	0.22
Rheumatoid arthritis	79 (1.42)	41 (1.16)	0.35	61 (1.45)	27 (1.13)	0.32
Charlson comorbidity index			0.51			0.98
0	287 (5.14)	178 (5.04)		195 (4.64)	115 (4.81)	
1	2,514 (45.05)	1,626 (46.08)		1,919 (45.66)	1,088 (45.50)	
2	1,638 (29.35)	986 (27.94)		1,209 (28.77)	682 (28.52)	
≥ 3	1,141 (20.45)	739 (20.94)		880 (20.94)	506 (21.16)	
Comedications						
Antihypertensive agents	2,990 (53.58)	1,985 (56.25)	0.01	2,255 (53.65)	1,317 (55.08)	0.27
Antihyperlipidemic agents	3,803 (68.15)	2,596 (73.56)	<0.001	2,828 (67.29)	1,751 (73.23)	<0.001
Antiplatelet agents	1,057 (18.94)	740 (20.97)	0.02	768 (18.27)	494 (20.66)	0.02
Anticoagulant agents	46 (0.82)	33 (0.94)	0.64	24 (0.57)	22 (0.92)	0.12
Body mass index (kg/m^2^)	28.04 ± 4.12	27.80 ± 3.95	0.01	28.03 ± 4.10	27.80 ± 3.92	0.04
Smoking status			0.99			0.82
Never smoker	2,913 (52.20)	1,841 (52.17)		2,206 (52.49)	1,236 (51.69)	
Former smoker	1,244 (22.29)	784 (22.22)		924 (21.98)	533 (22.29)	
Current smoker	1,423 (25.50)	904 (25.62)		1,073 (25.53)	622 (26.01)	
Family history						
Stroke	564 (10.11)	338 (9.58)	0.43	413 (9.83)	222 (9.28)	0.49
Heart disease	389 (6.97)	224 (6.35)	0.26	285 (6.78)	150 (6.27)	0.44
Health checkup data						
SBP (mmHg)	NA	NA	NA	128.17 ± 14.45	128.13 ± 14.23	0.92
FBG (mg/dL)	NA	NA	NA	138.44 ± 37.29	136.25 ± 33.08	0.05
LDL-C (mg/dL)	NA	NA	NA	113.72 ± 49.96	110.14 ± 40.49	<0.001
SCr (mg/dL)	NA	NA	NA	0.86 ± 0.22	0.86 ± 0.21	0.71

Data are presented as n (%) or mean ± SD.

^a^Household income was categorized into 20 classes, ranging from lowest (class 1) to highest (class 20), and further grouped into four income levels low (class 0–5), medium–low (class 6–10), medium–high (class 11–15), and high (class 16–20).

^b^Region of residence was classified as urban (Seoul, Busan, Daegu, Incheon, Gwangju, Daejeon, and Ulsan) or rural (Gyeonggi, Gangwon, Chungcheongbuk, Chungcheongnam, Jeollabuk, Jeollanam, Gyeongsangbuk, Gyeongsangnam, and Jeju).

^c^Microvascular complications of diabetes included diabetic retinopathy, diabetic neuropathy, and diabetic nephropathy.

Abbreviations: DAPA, dapagliflozin; EMPA, empagliflozin; MET, metformin; SD, standard deviation; BMI, body mass index; SBP, systolic blood pressure; LDL-C, low-density lipoprotein cholesterol; FBG, fasting blood glucose; SCr, serum creatinine; NA, not addressed

### Comparative risk of ischemic CVD events among patients using different SGLT-2is

A total of 55 and 10 composite ischemic CVD events occurred in the dapagliflozin and empagliflozin groups, respectively. In Models 1 and 2, patients newly treated with empagliflozin showed an approximately 55% lower risk of composite ischemic CVD events than those receiving dapagliflozin (aHR 0.42, 95% CI: 0.21–0.84; aHR 0.48, 95% CI: 0.23–0.98, respectively). However, in Model 3, after fully adjusting for all covariates, including clinical parameters—SBP, LDL-C, FBG, and SCr—the significant difference between empagliflozin and dapagliflozin groups was no longer observed (aHR 0.50, 95% CI: 0.24–1.03; Table 2). Additionally, across all three adjusted models, there were no significant differences between dapagliflozin and empagliflozin groups in the risk of individual components of composite ischemic CVD events, unstable angina, and all-cause mortality (Table 2).

**Table 2:** Risk comparison of ischemic CVD events between dapagliflozin and empagliflozin groups.

Outcomes	Event (%)	Crude model		Model 1^a^		Model 2^b^		Model 3^c^	
		Crude HR (95% CI)	*p*	Adjusted HR (95% CI)	*p*	Adjusted HR (95% CI)	*p*	Adjusted HR (95% CI)	*p*
**Composite ischemic CVD events**									
DAPA + MET	55 (0.99)	Reference		Reference		Reference		Reference	
EMPA + MET	10 (0.28)	**0.47 (0.24**–**0.92)**	**0.03**	**0.42 (0.21**–**0.84)**	**0.01**	**0.48 (0.23**–**0.98)**	**0.04**	0.50 (0.24–1.03)	0.06
**Myocardial infarction**									
DAPA + MET	12 (0.22)	Reference		Reference		Reference		Reference	
EMPA + MET	2 (0.06)	0.51 (0.11–2.36)	0.39	0.46 (0.10–2.12)	0.32	0.74 (0.14–4.02)	0.72	0.91 (0.16–5.13)	0.92
**Coronary revascularization**									
DAPA + MET	31 (0.56)	Reference		Reference		Reference		Reference	
EMPA + MET	8 (0.23)	0.65 (0.29–1.44)	0.29	0.60 (0.27–1.32)	0.20	0.65 (0.28–1.50)	0.31	0.72 (0.30–1.69)	0.44
**Ischemic stroke**									
DAPA + MET	20 (0.36)	Reference		Reference		Reference		Reference	
EMPA + MET	2 (0.06)	0.27 (0.06–1.18)	0.08	0.25 (0.06–1.07)	0.06	0.26 (0.06–1.18)	0.08	0.26 (0.06–1.20)	0.08
**Unstable angina**									
DAPA + MET	46 (0.82)	Reference		Reference		Reference		Reference	
EMPA + MET	13 (0.37)	0.83 (0.44–1.56)	0.56	0.77 (0.41–1.46)	0.42	0.76 (0.39–1.50)	0.43	0.71 (0.34–1.46)	0.35
**All-cause mortality**									
DAPA + MET	36 (0.65)	Reference		Reference		Reference		Reference	
EMPA + MET	13 (0.37)	1.15 (0.59–2.25)	0.69	1.02 (0.52–2.00)	0.95	0.78 (0.39–1.55)	0.48	0.50 (0.21–1.20)	0.12

Composite ischemic CVD events include myocardial infarction, coronary revascularization, or ischemic stroke. Bold values indicate statistical significance.

^a^Model 1 was adjusted for age and sex.

^b^Model 2 was adjusted for age, sex, metformin monotherapy duration, index year, household income, region of residence, comorbidities (hypertension, dyslipidemia, atrial fibrillation, chronic kidney disease, microvascular complications of diabetes [diabetic retinopathy, neuropathy, and nephropathy], and rheumatoid arthritis), CCI, comedications (antihypertensive, antihyperlipidemic, antiplatelet, and anticoagulant agents), BMI, smoking status, and family history of stroke or heart disease.

^c^Model 3 was adjusted for age, sex, metformin monotherapy duration, index year, household income, region of residence, comorbidities (hypertension, dyslipidemia, atrial fibrillation, chronic kidney disease, microvascular complications of diabetes [diabetic retinopathy, neuropathy, nephropathy], and rheumatoid arthritis), CCI, comedications (antihypertensive, antihyperlipidemic, antiplatelet, and anticoagulant agents), BMI, smoking status, family history of stroke or heart disease, systolic blood pressure, LDL-C, SCr, and FBG. Model 3 was adopted for the subgroup of people with available health checkup data (the total number of people was 4,203 for dapagliflozin and 2,391 for the empagliflozin group).

Abbreviations: DAPA, dapagliflozin; EMPA, empagliflozin; BMI, body mass index; CVD, cardiovascular disease; CCI, Charlson comorbidity index; CI, confidence interval; FBG, fasting blood glucose; HR, hazard ratio; SCr, serum creatinine; SD, standard deviation; LDL-C, low-density lipoprotein cholesterol

### Subgroup analysis

Subgroup analysis was conducted by stratifying patients based on age group, sex, BMI, FBG, eGFR, and LDL-C to assess the risk of composite ischemic CVD events. Both crude HR and aHR from Model 2 were reported. Overall, the results showed no significant differences between dapagliflozin and empagliflozin groups, except for patient group with eGFR levels between 60 and 90 mL/min/1.73m^2^ (S3 Table).

## Discussion

This retrospective cohort study assessed whether the risk of ischemic CVD events differed between dapagliflozin and empagliflozin, used as add-on therapies to metformin, among adults with T2DM. Using three stepwise-adjusted models, the results from Models 1 and 2 showed a potential difference in risk. However, in the fully adjusted Model 3, no significant difference in risk was observed between dapagliflozin and empagliflozin. Additionally, the two SGLT-2is showed no significant differences in the individual components of composite ischemic CVD events, unstable angina, and all-cause mortality in any of Models 1–3, supporting the conclusion that there were no statistically significant between-drug differences in reducing the incidence of ischemic CVD events.

In this study, Model 3 adjusted for key clinical parameters, including SBP, LDL-C, FBG, and SCr, in addition to traditional covariates such as comorbidities, an approach consistent with previous studies [19,21,44,45]. This approach was employed because the incidence of ischemic CVD events can be influenced by the presence of disease as well as by its severity, and incorporating these parameters allows for a more accurate reflection of the underlying disease state. These four clinical parameters are well-established risk factors for ischemic CVD events. SBP, a known risk factor for ischemic heart disease and stroke, has a documented positive association with the risk of both conditions [46,47]. LDL-C is supported by both clinical and genetic evidence as a correlate of atherosclerotic CVDs, including MI and stroke [48,49]. The atherogenic potential of LDL-C, such as its role in atherosclerotic plaque formation and acceleration of inflammatory processes, may contribute to ischemic CVD events [48]. Furthermore, impaired FBG level is a significant risk factor for ischemic CVD events, as abnormal glucose levels can induce endothelial dysfunction and promote plaque formation [50,51]. Decreased eGFR, as determined by creatinine levels, is associated with a higher risk of future ischemic CVD events [52,53]. High SCr levels are linked to endothelial dysfunction and contribute to increased cardiovascular risk by placing additional strain on the heart [54]. Therefore, by including these four significant risk factors as covariates, the present study offers more comprehensive and reliable findings on the development of ischemic CVD events.

Differences in the PK and PD properties of dapagliflozin and empagliflozin have been documented. Previous studies have reported that empagliflozin has greater selectivity for SGLT-2 over SGLT-1 and reaches peak concentration faster than dapagliflozin, whereas dapagliflozin has a slightly longer half-life, higher protein binding, and a larger volume of distribution [27,28]. However, differential effects on clinical parameters, such as HbA1c, FBG, and BMI, remain inconclusive [29,30,55,56]. Furthermore, it remains unclear whether differences in PK or PD properties translate into distinct effects on ischemic CVD events. Notably, a meta-regression analysis found no correlation between the magnitude of HbA1c reduction from SGLT-2is use and the incidence of CVD events [57]. These results imply that the development and manifestation of ischemic CVD events likely result from the complex interplay of multiple factors. In this context, the findings of current study—where significant differences in ischemic CVD events were observed between dapagliflozin and empagliflozin in Models 1 and 2, but not in Model 3 which adjusted for key clinical parameters—can be better understood.

Previous cohort studies have reported comparable effects of dapagliflozin and empagliflozin on subsequent cardiovascular outcomes [17–21]. While the results are similar, the current study differentiates itself in its design. Restricting the study population to patients receiving a combination of metformin and SGLT-2i provides a more controlled comparison between the two treatment groups. The type and number of antidiabetic agents used may reflect a patient’s glycemic control status or disease severity [23]. A previous study has shown that the duration for which HbA1c levels remain above target is positively associated with the number of antidiabetic agents used [58]. Additionally, different antidiabetic agents and their combinations may have varying effects on diabetes progression and subsequent ischemic CVD events [59]. For example, some antidiabetic agents, such as sulfonylureas, have been suggested to increase CVD risk patients with T2DM [60]. In this context, the current study focuses on patients who received dapagliflozin or empagliflozin as an add-on to metformin, offering more substantive evidence by comparing the distinct effects of these two SGLT-2is within a homogenous patient group—an approach that distinguishes it from previous studies.

This study has several key strengths. First, this study used comprehensive, nationwide healthcare data encompassing most of the South Korean population. Second, to enhance the robustness of the study, multivariable adjustments were performed incorporating health checkup data as covariates, including smoking status, family history of stroke or heart disease, and clinical parameters associated with ischemic CVD events [61,62]. By fully adjusting for potential risk factors for ischemic CVD events, this study effectively minimizes confounding effects.

Despite its strengths, this study has several limitations. First, because of the inherent limitations of claims and health checkup data, unmeasured or unknown residual confounding factors cannot be excluded. In particular, since information on HbA1c and diabetes duration was unavailable, data on FBG—the only available glycemic measure—were used for adjustment. However, considering that FBG may not fully reflect long-term glycemic control, residual confounding likely persisted and may have affected the results. Second, this study cannot rule out the possibility of selection bias. To ensure population homogeneity, individuals who had been treated with other antidiabetic agents within 1 year prior to the index date were excluded. This approach may have biased the study population toward patients with T2DM at earlier disease stages or those whose condition was relatively well controlled with metformin monotherapy. Moreover, the average participation rate in the general health checkup program during the study period was 75.4% [63]. Model 3 excluded individuals without health checkup data, which may have introduced bias related to data missing not at random (MNAR). Nevertheless, considering that even patients with early-stage diabetes have a higher risk for CVD and a greater need for risk factor management than the general population [64–67], examining CVD risk in this population still provides clinically meaningful insights. However, caution is warranted when interpreting and applying these findings. Third, a temporal difference in enrollment was observed between dapagliflozin and empagliflozin users, as empagliflozin was covered by the NHIS later than dapagliflozin in Korea. Although the index year was adjusted in the multivariate analyses in Models 2 and 3, the possibility that it may act as a time-related confounder cannot be ruled out. Finally, the small number of outcome events in this study may have limited its statistical power. However, the incidence of ischemic CVD events in Korean patients with T2DM has been reported to be lower than that in other countries [68–71], with previous Korean studies demonstrating event rates comparable to those observed in our study [18–20]. Moreover, the relatively healthy study population—consisting of patients with early-stage or well-controlled diabetes managed with metformin monotherapy, and a high proportion of patients in their 50s—may have contributed to the low event rates. Therefore, further studies with longer follow-up durations are warranted to address these limitations and improve the accuracy and generalizability of the findings.

In this nationwide cohort study, after adjustment for potential risk factors, including key clinical parameters, no significant difference in the risk of ischemic CVD events was observed between dapagliflozin and empagliflozin in combination with metformin in patients with T2DM. This finding provides real-world evidence that neither agent showed statistically significant superiority over the other in reducing the incidence of ischemic CVD events.

## Supporting information

S1 Table List of outcomes and comorbidities with corresponding codes. Abbreviations: ICD-10, International Classification of Diseases 10th revision.
(PDF)

S2 Table List of drugs used for the covariates with corresponding codes. Abbreviations: ACEI, angiotensin-converting enzyme inhibitors; ADP, adenosine diphosphate; ARB, angiotensin receptor blocker; BB, beta-blocker; CCB, calcium channel blocker; COX, cyclooxygenase; DU, diuretic; LMWH, low molecular weight heparin; PCSK9, proprotein convertase subtilisin/kexin type 9; PDE, phosphodiesterase; UFH, unfractionated heparin.
(PDF)

S3 Table Subgroup analysis for composite ischemic CVD events. Composite ischemic CVD events include MI, coronary revascularization, or ischemic stroke. Bold values indicate statistical significance. ^a^Hazard ratios were adjusted for age, sex, metformin monotherapy duration, index year, household income, region of residence, comorbidities (hypertension, dyslipidemia, atrial fibrillation, chronic kidney disease, microvascular complications of diabetes [diabetic retinopathy, neuropathy, and nephropathy], and rheumatoid arthritis), CCI, comedications (antihypertensive, antihyperlipidemic, antiplatelet, and anticoagulant agents), BMI, smoking status, and family history of stroke or heart disease. ^b^The BMI < 18.5 kg/m^2^ group (n = 9) and FBG < 70 mg/dL group (n = 3) are omitted because of the small total number of patients. Abbreviations: CVD, cardiovascular disease; DAPA, dapagliflozin; EMPA, empagliflozin; MET, metformin; BMI, body mass index; CCI, Charlson comorbidity index; CI, confidence interval; eGFR, estimated glomerular filtration rate; FBG, fasting blood glucose; HR, hazard ratio; LDL-C, low-density lipoprotein cholesterol; MI, myocardial infarction.
(PDF)
