# Supplementary material for: Comparison of ischemic cardiovascular events between dapagliflozin and empagliflozin in combination with metformin: A nationwide population-based cohort study
Source: PLoS One. 2025 Oct 16;20(10):e0333604. doi: 10.1371/journal.pone.0333604 (PMC12530601; doi:10.1371/journal.pone.0333604)
Supplement: S1 Table — Abbreviations: ICD-10, International Classification of Diseases 10th revision. (PDF) [file pone.0333604.s001.pdf]

S1 Table. List of outcomes and comorbidities with corresponding codes

Category	Corresponding codes
**Study outcomes**	
Myocardial infarction	ICD-10 codes I21–I23
Coronary revascularization	Procedure codes M6551, M6552, M6561, M6563, M6564, M6571, M6572, O1641, O1642, O1647, OA641, OA642, OA647
Ischemic stroke	ICD-10 codes I63
Angina pectoris	ICD-10 codes I20.0
**Comorbidities**	
Hypertension	ICD-10 codes I10–I15
Dyslipidemia	ICD-10 codes E78
Atrial fibrillation	ICD-10 codes I48
Chronic kidney disease	ICD-10 codes N18
Diabetic retinopathy	ICD-10 codes E10.3, E11.3, E12.3, E13.3, E14.3, H36.0
Diabetic neuropathy	ICD-10 codes E10.4, E11.4, E12.4, E13.4, E14.4, G63.2
Diabetic nephropathy	ICD-10 codes E10.2, E11.2, E12.2, E13.2, E14.2, N08.3
Rheumatoid arthritis	ICD-10 codes M05, M06, M08

Abbreviations: ICD-10, International Classification of Diseases 10th revision
