# Supplementary material for: Comparison of ischemic cardiovascular events between dapagliflozin and empagliflozin in combination with metformin: A nationwide population-based cohort study
Source: PLoS One. 2025 Oct 16;20(10):e0333604. doi: 10.1371/journal.pone.0333604 (PMC12530601; doi:10.1371/journal.pone.0333604)
Supplement: S2 Table — Abbreviations: ACEI, angiotensin-converting enzyme inhibitors; ADP, adenosine diphosphate; ARB, angiotensin receptor blocker; BB, beta-blocker; CCB, calcium channel blocker; COX, cyclooxygenase; DU, diuretic; LMWH, low molecular weight heparin; PCSK9, proprotein convertase subtilisin/kexin type 9; PDE, phosphodiesterase; UFH, unfractionated heparin. (PDF) [file pone.0333604.s002.pdf]

S2 Table. List of drugs used for the covariates with corresponding codes

Drug class		Drug name
Antihypertensive agents	ACEI	Benazepril, Captopril, Cilazapril, Enalapril, Enalaprilat, Fosinopril, Lisinopril, Moexipril, Perindopril, Quinapril, Ramipril, Trandolapril,
	ARB	Azilsartan, Candesartan, Eprosartan, Irbesartan, Losartan, Olmesartan, Telmisartan, Valsartan
	CCB	Amlodipine, Felodipine, Isradipine, Levamlodipine, Nicardipine, Nifedipine, Nimodipine, Nisoldipine, Diltiazem, Verapamil
	BB	Acebutolol, Atenolol, Betaxolol, Bisoprolol, Carvedilol, Esmolol, Labetalol, Metoprolol, Nadolol, Nebivolol, Pindolol, Propranolol
	Alpha-blocker	Doxazosin, Prazosin, Terazosin
	DU	Furosemide, Bumetanide, Torsemide, Triamterene, Spironolactone, Eplerenone, Hydrochlorothiazide, Chlorthalidone, Indapamide, Metolazone
	Others	Aliskiren, Clonidine, Hydralazine, Minoxidil
Antihyperlipidemic agents	Statin	Atorvastatin, Fluvastatin, Lovastatin, Pitavastatin, Pravastatin, Rosuvastatin, Simvastatin, Cerivastatin
	Fibrates	Fenofibrate, Gemfibrozil, Bezafibrate
	Niacin	Niacin
	Ezetimibe	Ezetimibe
	PCSK9 inhibitor	Alirocumab, Evolocumab

	Resin	Cholestyramine, Colestipol, Colesevelam
	Omega-3 fatty acid	Omega-3 fatty acid
Anticoagulant agents	Vitamin K antagonist	Warfarin
	UFH	Heparin
	LMWH	Dalteparin, Enoxaparin, Nadroparin
	Direct thrombin inhibitor	Dabigatran, Argatroban
	Factor Xa inhibitor	Rivaroxaban, Apixaban, Edoxaban, Fondaparinux
Antiplatelet agents	COX inhibitor	Aspirin, Triflusal, Indobufen
	PDE inhibitor	Cilostarzol, Dipyridamole
	P2Y12 inhibitor (ADP receptor inhibitor)	Clopidogrel, Ticlopidine, Prasugrel, Ticagrelor, Cangrelor

Abbreviations: ACEI, angiotensin-converting enzyme Inhibitors; ADP, adenosine diphosphate; ARB, angiotensin receptor blocker; BB, beta-blocker; CCB, calcium channel blocker; COX, cyclooxygenase; DU, diuretic; LMWH, low molecular weight heparin; PCSK9, proprotein convertase subtilisin/kexin type 9; PDE, phosphodiesterase; UFH, unfractionated heparin
