# Supplementary material for: Comparison of ischemic cardiovascular events between dapagliflozin and empagliflozin in combination with metformin: A nationwide population-based cohort study
Source: PLoS One. 2025 Oct 16;20(10):e0333604. doi: 10.1371/journal.pone.0333604 (PMC12530601; doi:10.1371/journal.pone.0333604)
Supplement: S3 Table — Composite ischemic CVD events include MI, coronary revascularization, or ischemic stroke. Bold values indicate statistical significance. aHazard ratios were adjusted for age, sex, metformin monotherapy duration, index year, household income, region of residence, comorbidities (hypertension, dyslipidemia, atrial fibrillation, chronic kidney disease, microvascular complications of diabetes [diabetic retinopathy, neuropathy, and nephropathy], and rheumatoid arthritis), CCI, comedications (antihypertensive, antihyperlipidemic, antiplatelet, and anticoagulant agents), BMI, smoking status, and family history of stroke or heart disease. bThe BMI < 18.5 kg/m2 group (n = 9) and FBG < 70 mg/dL group (n = 3) are omitted because of the small total number of patients. Abbreviations: CVD, cardiovascular disease; DAPA, dapagliflozin; EMPA, empagliflozin; MET, metformin; BMI, body mass index; CCI, Charlson comorbidity index; CI, confidence interval; eGFR, estimated glomerular filtration rate; FBG, fasting blood glucose; HR, hazard ratio; LDL-C, low-density lipoprotein cholesterol; MI, myocardial infarction. (PDF) [file pone.0333604.s003.pdf]

S3 Table. Subgroup analysis for composite ischemic CVD events

Variable	Study group	No.	Event (%)	Crude HR (95% CI)	*p*	Adjusted HR[a] (95% CI)	*p*
Age (years)							
<65	DAPA + MET	4,925	44 (0.89)	Reference		Reference	
	EMPA + MET	3,038	9 (0.30)	0.51 (0.25–1.06)	0.07	0.54 (0.25–1.18)	0.12
≥65	DAPA + MET	655	11 (1.68)	Reference		Reference	
	EMPA + MET	491	1 (0.20)	0.25 (0.03–2.02)	0.20	0.18 (0.02–2.05)	0.17
Sex							
Men	DAPA + MET	3,226	43 (1.33)	Reference		Reference	
	EMPA + MET	2,077	9 (0.43)	0.50 (0.24–1.03)	0.06	0.53 (0.24–1.15)	0.11
Women	DAPA + MET	2,354	12 (0.51)	Reference		Reference	
	EMPA + MET	1,452	1 (0.07)	0.26 (0.03–2.00)	0.19	0.15 (0.02–1.35)	0.09
BMI[b] (kg/m^2)							
18.5–24.9	DAPA + MET	1,225	18 (1.47)	Reference		Reference	
	EMPA + MET	821	2 (0.24)	0.33 (0.08–1.46)	0.14	0.32 (0.07–1.54)	0.15
25–29.9	DAPA + MET	2,834	26 (0.92)	Reference		Reference	

	EMPA + MET	1,836	6 (0.33)	0.51 (0.21–1.25)	0.14	0.48 (0.19–1.25)	0.13
≥30	DAPA + MET	1,514	11 (0.73)	Reference		Reference	
	EMPA + MET	870	2 (0.23)	0.54 (0.12–2.50)	0.43	0.97 (0.16–5.86)	0.97
FBG[b] (mg/dL)							
70–99.9	DAPA + MET	339	6 (1.77)	Reference		Reference	
	EMPA + MET	200	1 (0.50)	0.57 (0.07–5.03)	0.61	0.63 (0.01–27.98)	0.81
100–125.9	DAPA + MET	1,867	17 (0.91)	Reference		Reference	
	EMPA + MET	1,288	2 (0.16)	0.30 (0.07–1.33)	0.11	0.42 (0.09–2.06)	0.28
≥126	DAPA + MET	3,363	32 (0.95)	Reference		Reference	
	EMPA + MET	2,030	7 (0.34)	0.55 (0.24–1.25)	0.15	0.47 (0.19–1.16)	0.10
eGFR (mL/min/1.73 m^2)							
<60	DAPA + MET	162	2 (1.23)	Reference		Reference	
	EMPA + MET	107	2 (1.87)	3.17 (0.29–34.97)	0.35	NA	1.00
60–89.9	DAPA + MET	2,690	34 (1.26)	Reference		Reference	
	EMPA + MET	1,679	5 (0.30)	**0.35 (0.13–0.89)**	**0.03**	**0.33 (0.12–0.91)**	**0.03**
≥90	DAPA + MET	2,702	19 (0.70)	Reference		Reference	
	EMPA + MET	1,721	3 (0.17)	0.49 (0.14–1.69)	0.26	0.45 (0.12–1.67)	0.23

LDL-C (mg/dL)							
<70	DAPA + MET	618	12 (1.94)	Reference		Reference	
	EMPA + MET	380	1 (0.26)	0.21 (0.03–1.63)	0.14	0.22 (0.02–2.64)	0.23
70–99.9	DAPA + MET	1,029	15 (1.46)	Reference		Reference	
	EMPA + MET	641	4 (0.62)	0.65 (0.21–2.01)	0.46	0.35 (0.09–1.32)	0.12
100–129.9	DAPA + MET	1,175	15 (1.28)	Reference		Reference	
	EMPA + MET	658	2 (0.30)	0.36 (0.08–1.59)	0.18	0.31 (0.06–1.79)	0.19
130–159.9	DAPA + MET	824	5 (0.61)	Reference		Reference	
	EMPA + MET	428	2 (0.47)	1.19 (0.23–6.25)	0.84	NA	1.00
≥160	DAPA + MET	557	6 (1.08)	Reference		Reference	
	EMPA + MET	284	1 (0.35)	0.50 (0.06–4.29)	0.53	1.71 (0.04–71.41)	0.78

Composite ischemic CVD events include MI, coronary revascularization, or ischemic stroke. Bold values indicate statistical significance.

[a]Hazard ratios were adjusted for age, sex, metformin monotherapy duration, index year, household income, region of residence, comorbidities (hypertension, dyslipidemia, atrial fibrillation, chronic kidney disease, microvascular complications of diabetes [diabetic retinopathy, neuropathy, and nephropathy], and rheumatoid arthritis), CCI, comedications (antihypertensive, antihyperlipidemic, antiplatelet, and anticoagulant agents), BMI, smoking status, and family history of stroke or heart disease.

[b]The BMI <18.5 kg/m^2 group (n = 9) and FBG <70 mg/dL group (n = 3) are omitted because of the small total number of patients.

Abbreviations: CVD, cardiovascular disease; DAPA, dapagliflozin; EMPA, empagliflozin; MET, metformin; BMI, body mass index; CCI, Charlson comorbidity index; CI, confidence interval; eGFR, estimated glomerular filtration rate; FBG, fasting blood glucose; HR, hazard ratio; LDL-C, low-density lipoprotein cholesterol; MI, myocardial infarction.
